# Paresthesia and Facial Bone Destruction due to Chronic Granulomatous Invasive Aspergillosis in the Left Maxillary Sinus

**DOI:** 10.1002/ccr3.72477

**Published:** 2026-04-03

**Authors:** Rawa Badri, Matthew C. Fisher, Ahmed Hassan Fahal

**Affiliations:** ^1^ The Mycetoma Research Centre University of Khartoum Khartoum Sudan; ^2^ Department of Infectious Disease Epidemiology, School of Public Health Imperial College London London UK

**Keywords:** antifungal therapy, bone destruction, invasive aspergillosis, maxillary sinus, paresthesia

## Abstract

We report a case of chronic granulomatous invasive aspergillosis in a 56‐year‐old male teacher from South Kordofan, Sudan, who underwent surgery in 2007 to remove his left orbital aspergillus granuloma. The patient developed progressive facial swelling beginning in 2017 and paraesthesia in the left zygomatico‐maxillary region, despite initial treatment. A soft tissue mass in the left maxillary sinus and widespread bone loss affecting the zygomatic arch, maxillary antrum, and fronto‐temporal lytic lesions were seen on computed tomography. Histopathological analysis confirmed the recurrence of aspergillosis. Itraconazole therapy was administered to the patient after surgical excision, although treatment compliance was subpar. A subsequent wide local excision was performed in 2021, but the patient developed post‐operative complications, including wound infection and dysphagia, leading to death four days after surgery. This case highlights the aggressive nature of chronic invasive aspergillosis, the importance of adequate initial treatment duration, and the potential for devastating complications, including facial bone destruction and neurological involvement manifesting as paresthesia.

## Key Clinical Message

Chronic Granulomatous Invasive Aspergillosis can pursue an aggressive course even in immunocompetent patients, and long‐term antifungal therapy with strict adherence following complete surgical excision is essential to prevent devastating recurrence with neurological and bony complications.

## 1 Introduction

Invasive aspergillosis is a rare opportunistic fungal infection that typically affects immunocompromised patients [1, 2]. The lung is the most common site of infection, but other body sites can serve as foci, such as the paranasal sinuses [2]. If the primary infection is not controlled, tissue invasion and hematogenous spread to other organs can occur, resulting in invasive aspergillosis [2]. Aspergillus infection of the maxillary sinus can be an invasive or noninvasive infection [3]. The noninvasive infection is similar to chronic bacterial sinusitis in symptoms, clinical signs, and X‐ray findings. In the invasive form, bone destruction and orbital involvement can occur, and it is similar to a malignant tumor [4]. Nasosinus aspergillosis occurs in Sudan, where females are predominantly affected [5]. The commonest mode of clinical presentation is nasal polyposis. The ethmoid sinus is the most affected, followed by the maxillary and frontal sinuses. It has been reported that aspergillosis is the commonest cause of noncongenital unilateral proptosis in Sudan [5]. The paranasal sinuses are the usual portal of entry for the organism; orbital extension is more common in immunocompromised individuals [6]. The diseases of the sinuses range from the common allergic rhinitis to fungal antigens, to allergic Aspergillus sinusitis, to fungus balls, to invasive and fulminant fungal sinusitis capable of causing death within hours to days [7].

Invasive sinus aspergillosis results from inhalation of ubiquitous Aspergillus conidia (most commonly *A. fumigatus* and *A. flavus* ) that colonize the paranasal sinuses and, in the setting of impaired host immunity, invade the mucosa, submucosa, blood vessels, and bone through angioinvasive hyphal growth, leading to tissue necrosis and thrombosis [8, 9]. The disease has predominantly been described in immunocompromised individuals, such as those with hematologic malignancies, prolonged neutropenia, solid organ transplant recipients, those using corticosteroids, and those with uncontrolled diabetes. However, cases in immunocompetent individuals, particularly in South Asia and the Middle East, have also been reported [10, 11]. Three clinicopathological subtypes are described: acute fulminant (progressing within days to weeks), chronic invasive, and granulomatous invasive [8]. The clinical features include fever resistant to antibiotics, nasal congestion, facial pain, periorbital swelling, proptosis, and cranial nerve palsies if orbital or intracranial extension is present, and mortality for acute fulminant disease is 50%–80% [12, 13]. The diagnostic features include CT and MRI to show bony erosion and soft‐tissue extension, nasal endoscopy, tissue biopsy showing septate, acute‐angle branching hyphae on histopathology, and positive fungal culture results. Galactomannan antigen may be used as supportive evidence [8, 14]. Management demands a multidisciplinary approach comprising urgent surgical debridement, systemic antifungal therapy (voriconazole or liposomal amphotericin B as first‐line agents), and reversal of the underlying immunosuppression [12, 15].

Early diagnosis and treatment of the infection reduces mortality from disseminated aspergillosis. Radiological techniques are advocated for early diagnosis of infection, but clinical detection remains the fundamental method for establishing the diagnosis [16].

## 2 Case History and Examination

The patient was a 56‐year‐old male teacher from South Kordofan (Aldalang) who was referred from Aldalang Dental Hospital to Khartoum Teaching Dental Hospital, Khartoum, Sudan, in October 2018 for a swelling on the left side of the face. The swelling began in 2017 and gradually increased in size. The patient had eye surgery in 2007 for the removal of the left orbital aspergillus granuloma, and he took Itraconazole capsules. He had an one‐day hospital admission after the operation. On examination, there was tenderness in the lateral side of the left eye, cheek, and zygomatico‐maxillary region, and paresthesia at the left zygomatico‐maxillary region. The patient was not pale, jaundiced, or cyanosed. A head and neck examination revealed limitations in mouth opening. Intra‐oral examination revealed a normal soft palate, tonsils, floor of the mouth, and gingiva. There was mucosal swelling in the left cheek against the 2nd and 3rd molars. Examination of the mandible and hard palate revealed no abnormality. There was a left maxillary swelling upon examination of the Maxilla. The swelling was extending from the left lower temporal region and medial canthus of the eye superiorly to the left lateral angle of the mouth and the tragus of the left ear inferiorly, extra‐orally, and from the lateral tooth to the second molar intra‐orally. The swelling has areas of firmness and fluctuation.

## 3 Diagnostic Assessment

**FIGURE 1:**
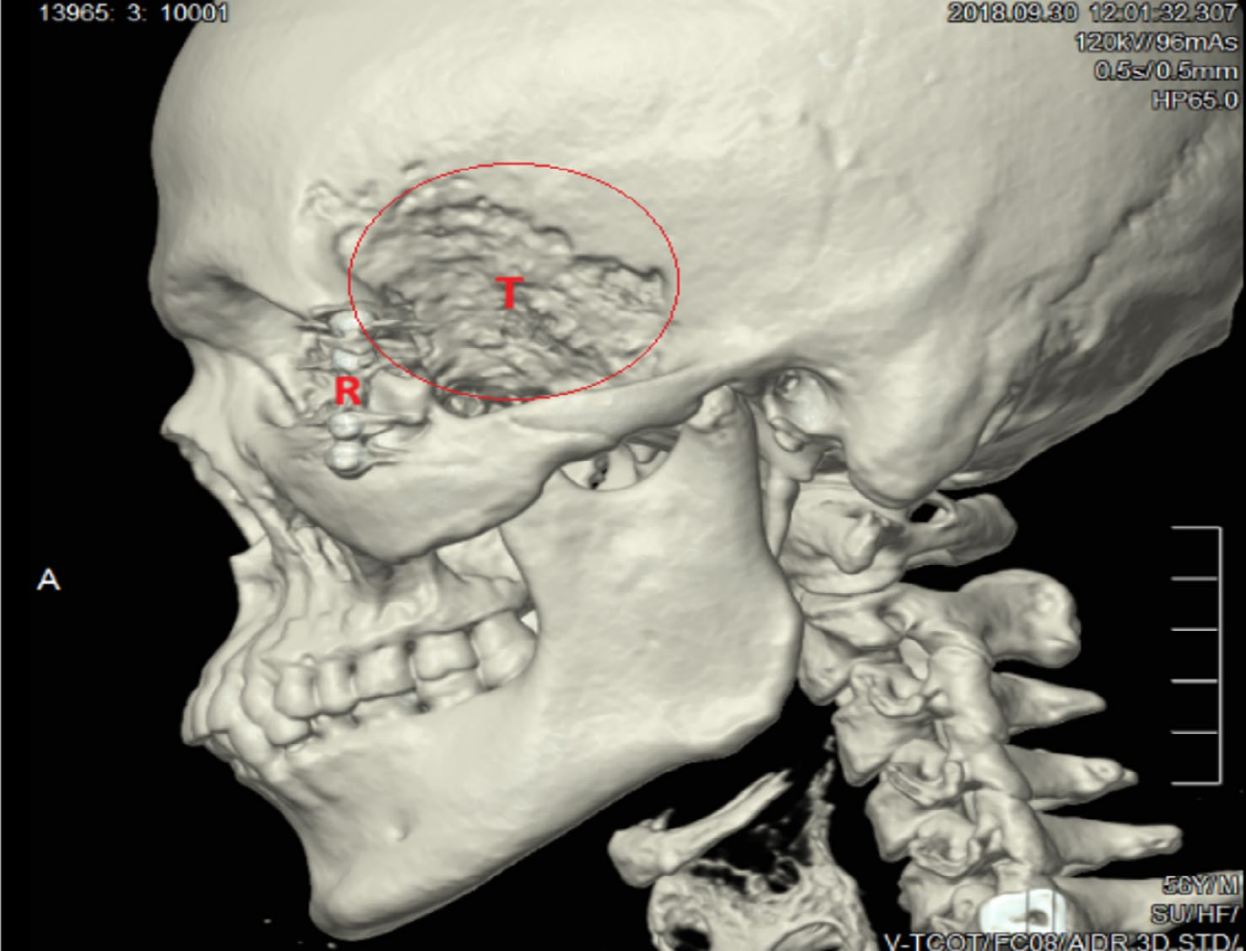
Computed tomography (CT) scan of the facial skeleton showing (R) the internal fixation at the lateral rim of the left bony orbit and (T) left fronto‐temporal lytic lesions.

**FIGURE 2:**
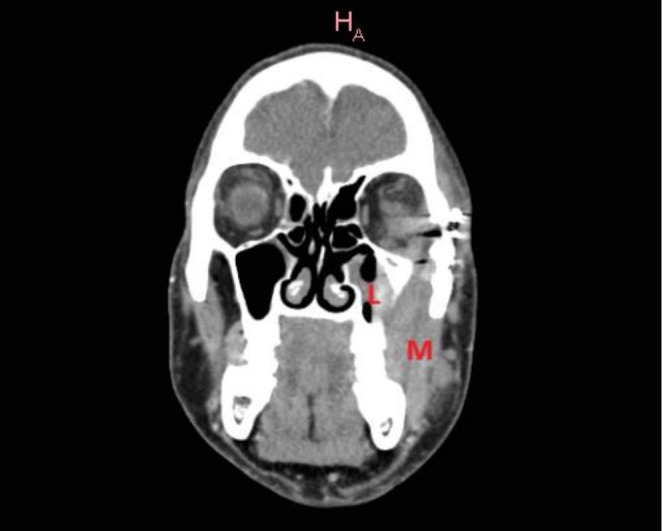
Computed tomography (CT) scan shows (L) a left maxillary soft tissue mass and (M) enlargement of the masticator space.

**FIGURE 3:**
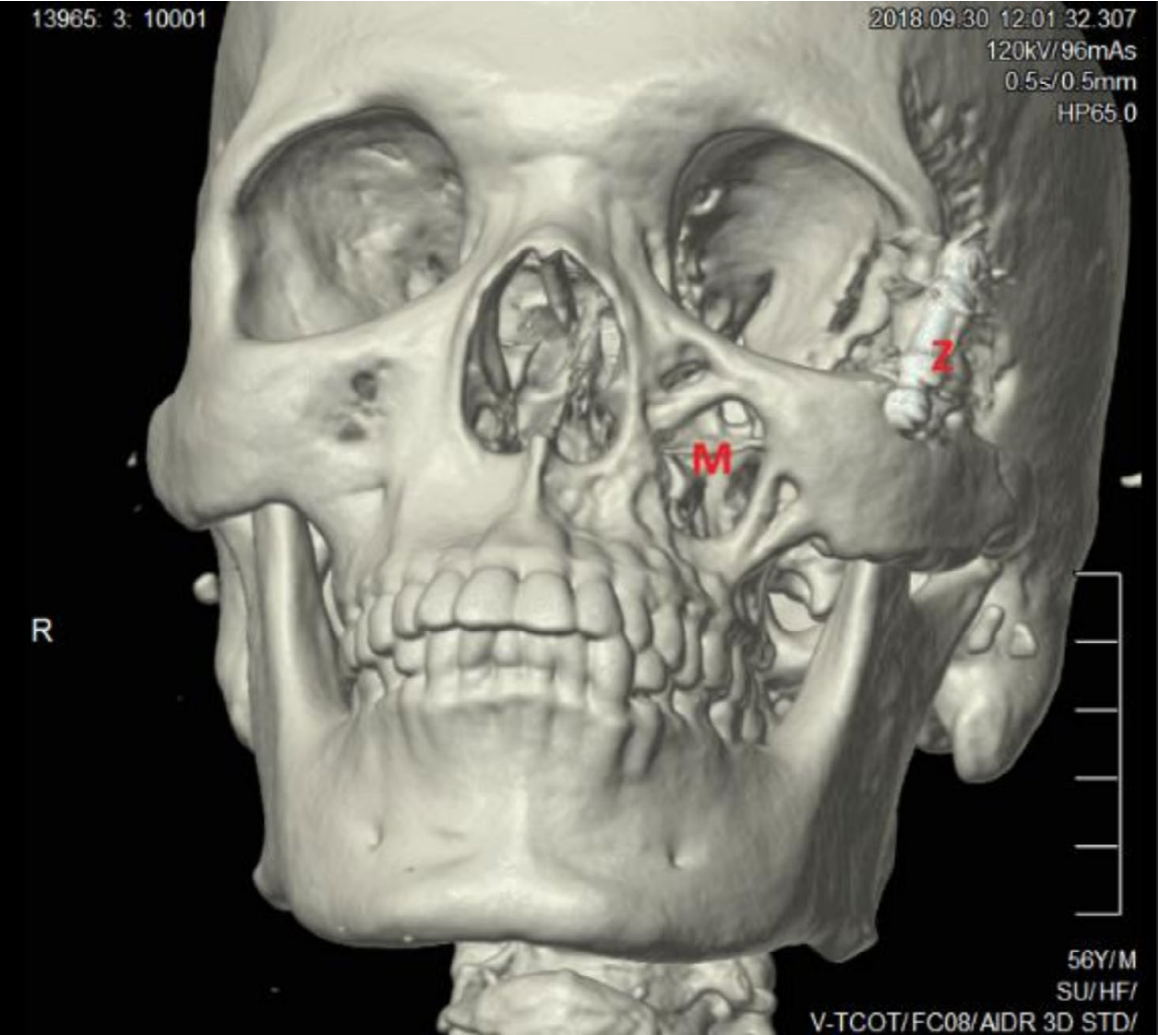
Facial skeleton computed tomography (CT) scan shows destruction of bone involving the (Z) zygomatic arch and the (M) anterior surface of the left maxillary antrum.

Chest X‐ray was normal. A computed Tomography (CT) scan of the facial skeleton showed internal fixation at the lateral rim of the left bony orbit and left fronto‐temporal lytic lesions (Figure 1). The CT scan also showed a left maxillary soft tissue mass, enlargement of the masticator space (Figure 2), and destruction of bone involving the zygomatic arch and the anterior surface of the left maxillary antrum (Figure 3).

**FIGURE 4:**
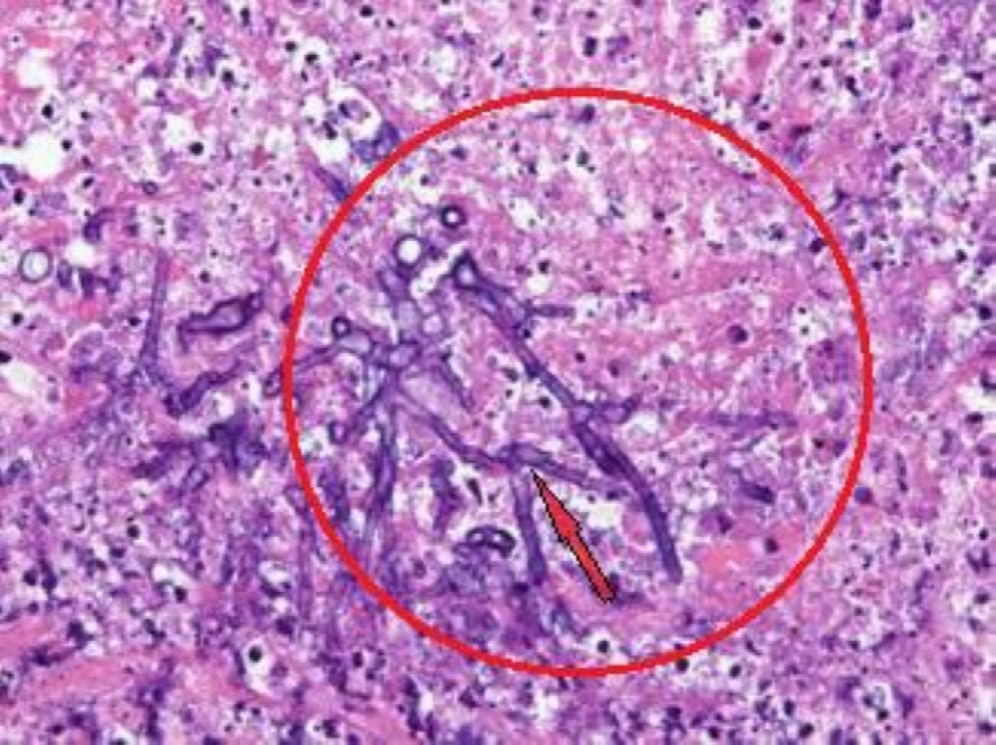
Invasive fungal sinusitis. Necrotic tissue with abundant *Aspergillus* hyphae with characteristic angular branching and septation [17].

A biopsy was taken, and histopathological examination revealed aspergillosis (granulomatous inflammation/aspergillus granuloma) (Figure 4) [17]. Renal function test within normal limits; the patient also has a normal complete blood count. The electrocardiogram and Echocardiogram were normal. The viral screening blood test was negative for HIV, HBV, and HCV.

## 4 Therapeutic Intervention

Hypertension was detected before surgery, and the patient received Amlodipine. The patient was admitted to the hospital, and surgical excision of the aspergillus granuloma was done under general anesthesia on November 18, 2018. He was discharged on November 25, 2018.

**FIGURE 5:**
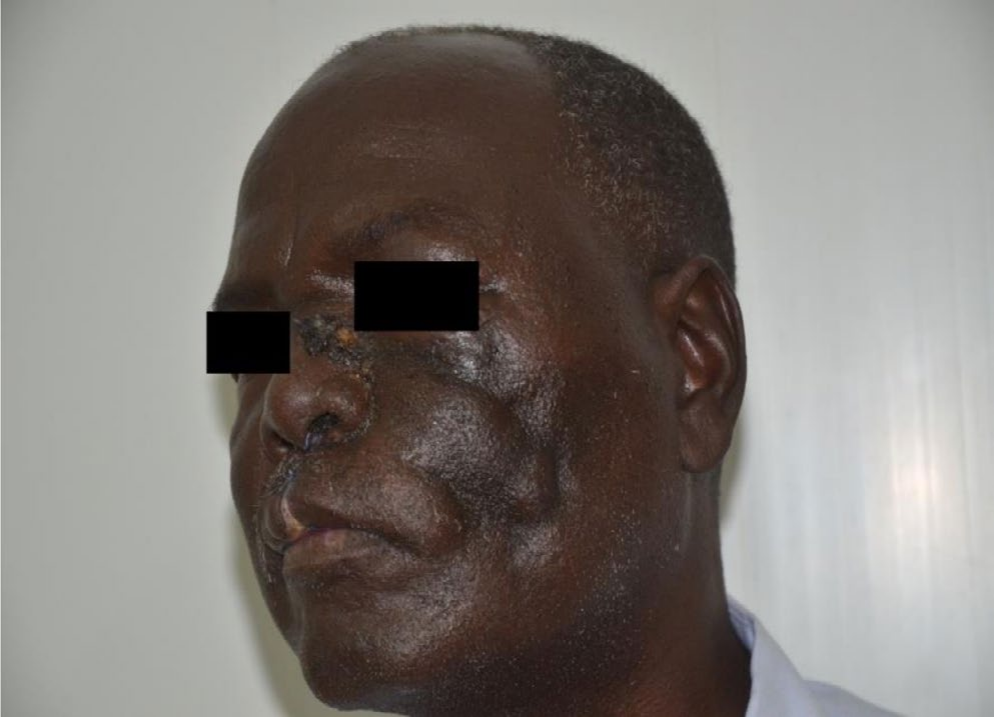
Photograph of the patient shows the surgical scar extending from near the medial canthus of the left eye down the lateral side of the nose to the middle of the upper and a swelling measuring 6 × 4 cm at the left fronto‐temporal region.

In December 2018, the patient presented to us at the Mycetoma Research Centre in Khartoum, Sudan. On examination, the patient was not jaundiced, pale, or cyanosed. There was a surgical scar extending from near the medial canthus of the left eye down the lateral side of the nose to the middle of the upper lip. Also, a swelling measuring 6 × 4 cm was noted in the left frontotemporal region, caused by the temporal residual of the aspergillosis (Figure 5). The patient complained of excessive salivation following the surgical operation.

## 5 Conclusions and Results

Itraconazole 200 mg BD was prescribed for the patient following his presentation to the Mycetoma Research Centre in December 2018; however, treatment adherence was poor. The patient was subsequently lost to follow‐up until 2021, when, according to information obtained retrospectively from the patient's caregiver, he presented to Khartoum Dental Hospital and underwent wide local excision of the recurrent Aspergillus granuloma. The patient's caregiver reported that the wound became infected and the patient experienced dysphagia a few days after the operation, necessitating nasogastric tube insertion. The patient died four days postoperatively. The patient's caregiver did not report the cause of death, and it could not be formally established; however, given the documented post‐operative wound infection, sepsis remains a possible contributing mechanism. Table 1 summarizes the clinical timeline, management, and antifungal adherence across treating institutions.

**TABLE 1:** Clinical timeline, management, and antifungal adherence across treating institutions.

Facility	Date	Management provided	Antifungal adherence duration
Aldalang dental hospital	2007	Surgical excision of left orbital aspergillus granuloma; short‐term oral antifungal Itraconazole	Short‐term (not specified)
Khartoum teaching dental hospital	November 2018	Surgical excision of left maxillary aspergillus granuloma under general anesthesia.	
Mycetoma research centre	December 2018	Prescribed Itraconazole 200 mg BD.	1 year without adherence
Khartoum dental hospital	2021	Wide local excision of recurrent aspergillus granuloma.	

## 6 Case Discussion

Mycotic infections of the paranasal sinuses are common in apparently healthy patients. However, many conditions favor fungal infections, such as diabetes, long‐term treatments (antibiotics and corticosteroids), radiotherapy and chemotherapy, immunosuppressive treatments, and immunodeficiency [18]. Fulminant aspergillosis must be differentiated from mucormycosis, pseudomonas orofacial lesions, or Wegener's granulomatosis [19]. In this case report, mucormycosis was excluded based on the histopathological confirmation of aspergillosis. Wegener's granulomatosis was excluded based on the absence of systemic vasculitis features and normal renal function. Fungal culture, PCR, molecular diagnostic, and serological testing to identify the Aspergillus species were not performed, as these resources were unavailable at the treating institution where the biopsy was taken, which represents a limitation of this case report. Treatment of paranasal sinus aspergillosis consists of surgical removal of the diseased mucosa with sinus drainage and adequate aeration of the sinus [20]. Medical treatment complements surgical treatment of invasive aspergillosis. Liposomal amphotericin B is effective for treating invasive aspergillosis sinusitis; voriconazole is also effective, with greater efficacy and less toxicity [6, 19]. Surgical intervention is particularly urgent in orbital aspergillosis when vision is still preserved [21].

The clinical course in this case deviates significantly from the acute presentations of invasive fungal rhinosinusitis (AIFRS) typically documented in the literature. A recent systematic review of 387 cases indicates that AIFRS generally affects younger populations (mean age 32.5 for *Aspergillus*) and progresses rapidly within four weeks, primarily in immunocompromised hosts [22]. In contrast, our patient exhibited a chronic, destructive granulomatous variant over several years, which is more characteristic of infections in immunocompetent individuals in specific geographical regions. Furthermore, while contemporary reports highlight high efficacy rates (89.47%) for newer triazoles such as isavuconazole [22], our case demonstrates that even gold‐standard therapy is secondary to adherence to treatment. According to the IDSA guidelines [15], a minimum of 6 to 12 weeks of antifungal induction is required; however, the ‘subpar’ compliance and three‐year lapse in follow‐up observed here allowed for the aggressive ‘centrifugal’ spread that ultimately led to a fatal outcome.

Although invasive fungal sinusitis has been documented in subtropical regions such as North Africa and Southeast Asia, recent literature reports its rare occurrence in non‐endemic regions, such as the Balkans [23]. The study described a 40‐year‐old immunocompetent male with a 5‐year history of recurrent nasal polyposis, a clinical course that closely parallels the long‐lasting, indolent progression observed in our patient. In their report, the disease eventually manifested as sudden proptosis due to erosion of the ethmoid bone and orbital expansion, mirroring the aggressive ‘centrifugal’ spread and bone destruction seen in our case [23]. However, unlike our patient's fatal outcome following ‘subpar’ adherence, the case from the Balkans demonstrates a successful prognosis achieved through a combination of radical surgery and a defined nine‐month course of voriconazole. This comparison further validates that, while surgical debridement is essential, strict adherence to prolonged, multi‐month systemic antifungal protocols is the definitive factor in arresting the progression of Chronic Granulomatous Invasive Fungal Rhinosinusitis (CGIFRS).

**FIGURE 6:**
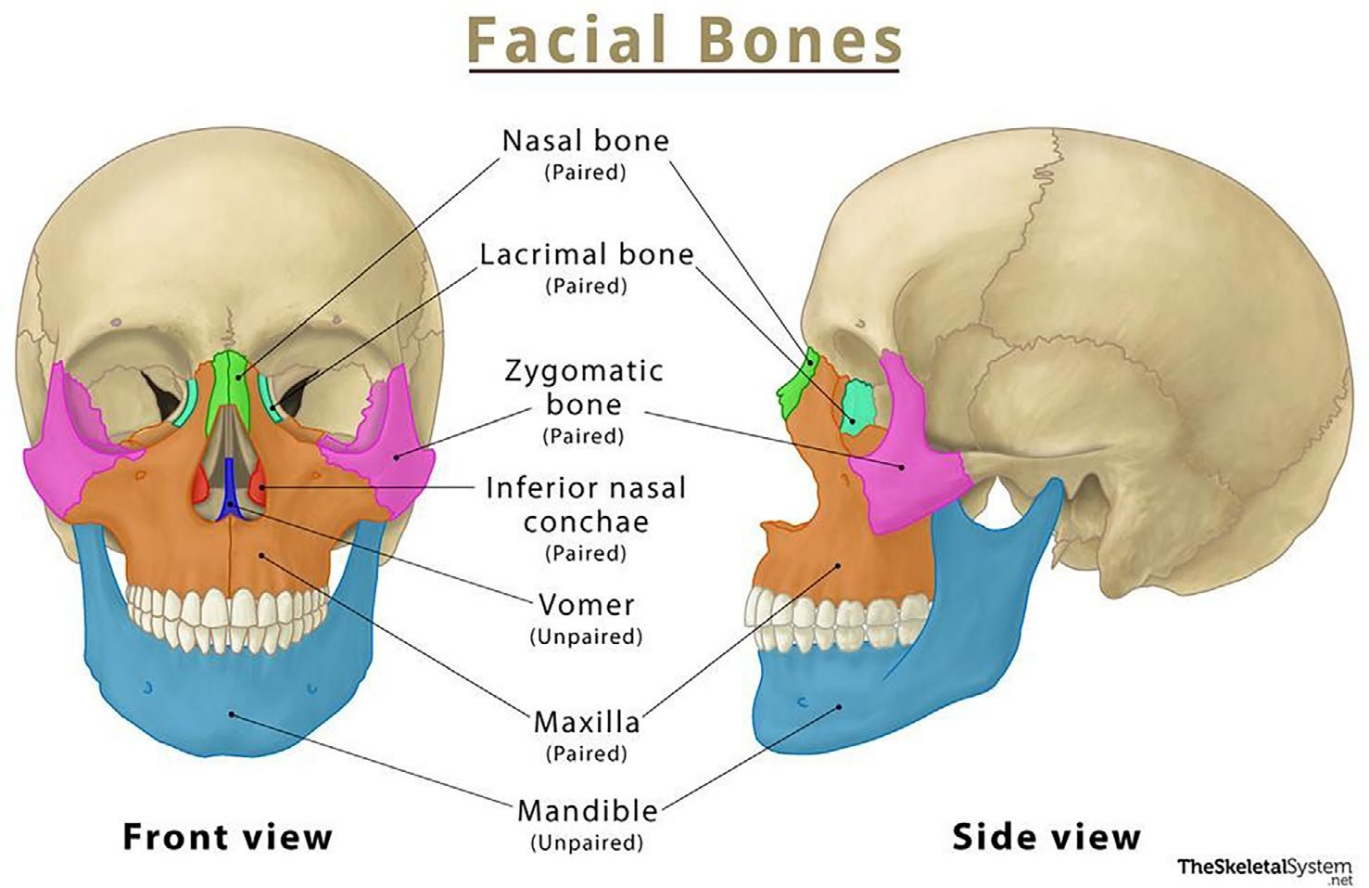
Normal anatomical reference of the facial skeleton. Frontal and lateral views highlighting the maxilla (the site of the primary mass and antral destruction) and the zygomatic bone (the site of the arch discontinuity seen in Figure 3) [24].

The late presentation of this patient, which led to the destruction of the maxillary and the zygomatic arch and the lytic lesions on the fronto‐temporal region, is due to the absence of pain during the progression of the disease; hence, he didn't go to the hospital until the swelling had become apparent and caused left eye proptosis. After the first surgical excision of the orbital aspergillus granuloma, the patient received medical treatment for a short period. Then the patient presented again after years with tenderness, paresthesia, and swelling in the left side of the face due to a recurrent aspergillus granuloma in the left maxillary sinus, which may have resulted from the inadequate duration of medical treatment following the first surgery, which was insufficient to eradicate the fungal infection. Alternatively, an extension of the maxillary sinus aspergillus granuloma may have been present from the outset of the disease. However, it may not have been apparent or detectable at his initial presentation. Paresthesia of the zygomaticomaxillary region, although uncommon, is a significant manifestation of Chronic Granulomatous Invasive Aspergillosis and points to the involvement of the maxillary nerve by the destructive process. The extensive bone destruction observed on CT scans, affecting the zygomatic arch, maxillary antrum, and fronto‐temporal regions, is characteristic of invasive aspergillosis and distinguishes this condition from other noninvasive aspergillosis infections. Together, these features underscore the consequences of delayed diagnosis and inadequate antifungal therapy. The aggressive nature of the infection is highlighted when comparing the patient's bone loss (Figure 3) to normal facial anatomy (Figure 6) [24]. The destruction of the maxillary antrum and zygomatic arch directly corresponds to the maxillary nerve's pathway, explaining the patient's paresthesia.

Early diagnosis of invasive fungal infections (IFIs) is essential for improving patient outcomes; however, it remains challenging due to nonspecific clinical presentations and the limited sensitivity and specificity of current diagnostic tests [25]. Recent advances have enabled better early detection through molecular diagnostics and nonculture‐based technologies, allowing quicker identification of pathogens and resistance [26]. The most effective approach to treating invasive and recurrent aspergillosis includes mold‐active azoles, with voriconazole and isavuconazole being first‐line agents [27]. Posaconazole should be used for prophylaxis in high‐risk patients [27], whereas for salvage and recurrent aspergillosis, lipid formulations of amphotericin B, including liposomal amphotericin B, should be used, with consideration of combination antifungal therapy [28]. No RCTs have established the optimum duration for invasive sinus aspergillosis. Guidelines recommend debridement and systemic antifungals (voriconazole is the first choice), and this is individualized based on clinical/radiological responses and immune status [15, 29]. Case series describe azole therapy for 6–12 months for chronic invasive disease [30, 31]. Acute forms in immunocompromised patients required a median of 82 days in one analysis [32]. For recurrence prevention post‐surgery, 6 weeks of itraconazole may reduce relapse [33].

Olorofim, a first‐in‐class orotomide, exhibits potent activity against refractory and azole‐resistant *Aspergillus* species [26]. Rapid clinical and radiologic improvements in uncontrolled infections support its role in managing severe invasive cases [26, 34].

## Author Contributions

**Rawa Badri:** conceptualization, writing – original draft, writing – review and editing. **Matthew C. Fisher:** supervision, writing – review and editing. **Ahmed Hassan Fahal:** supervision, writing – review and editing.

## Funding

The authors have nothing to report.

## Consent

Written informed consent was obtained from the patient's next of kin, as the patient is deceased, to publish this report in accordance with the journal's patient consent policy.

## Conflicts of Interest

The authors declare no conflicts of interest.

## Data Availability

Data sharing not applicable to this article as no datasets were generated or analysed during the current study.
